# The motherhood choices decision aid for women with rheumatoid arthritis increases knowledge and reduces decisional conflict: a randomized controlled trial

**DOI:** 10.1186/s12891-015-0713-0

**Published:** 2015-09-22

**Authors:** T. Meade, E. Dowswell, N. Manolios, L. Sharpe

**Affiliations:** School of Social Sciences and Psychology, Western Sydney University, PO BOX 1797, Penrith, NSW, 2751 Australia; Western Clinical School, Faculty of Medicine, The University of Sydney, Sydney, NSW, 2006 Australia; School of Psychology, The University of Sydney, Sydney, NSW, 2006 Australia

## Abstract

**Background:**

For many women with Rheumatoid Arthritis (RA) motherhood decisions are complicated by their condition and complex pharmacological treatments. Decisions about having children or expanding their family require relevant knowledge and consultation with their family and physician as conception and pregnancy has to be managed within the RA context. Relevant information is not readily available to women with RA. Therefore a randomized controlled study was conducted to evaluate the effectiveness of a new motherhood decision aid (DA) developed specifically for women with RA.

**Methods:**

One hundred and forty-four women were randomly allocated to either an intervention or control group. All women completed a battery of questionnaires at pre-intervention, including, the Pregnancy in Rheumatoid Arthritis Questionnaire (PiRAQ), the Decisional Conflict Scale (DCS), the Hospital Anxiety and Depression Scale (HADS), and the Arthritis Self-Efficacy Scale (ASES), and provided basic demographic information. Women in the DA group were sent an electronic version of the DA, and completed the battery of questionnaires for a second time post-intervention.

**Results:**

Women who received the DA had a 13 % increase in relevant knowledge (PiRAQ) scores and a 15 % decrease in scores on the decisional conflict (DCS), compared to the control group (1 %, 2 % respectively). No adverse psychological effects were detected as evident in unchanged levels of depression and anxiety symptoms.

**Conclusions:**

The findings of this study suggest that this DA may be an effective tool in assisting women with RA when contemplating having children or more children.

**Trial registration:**

Australian New Zealand Clinical Trials Registry, http://www.anzctr.org.au/, ACTRN12615000523505.

## Background

The decision to have children is influenced by a number of factors including relationship quality and readiness, education level, financial considerations, and the availability of social support [1–3]. Women with chronic medical conditions may experience additional challenges associated with their health care needs, social stigma pertaining to their parenting capacity, and the impact of their condition on pregnancy and motherhood [4–7].

While parenting is generally accepted as a positive experience, for many women with chronic conditions such as Rheumatoid Arthritis (RA), the physical symptoms can significantly impair their role and performance as mothers [8–10]. Some women experience feelings of guilt and shame over the impact of their illness on parenting and have to adjust their expectations of themselves as mothers [11, 12].

RA is a chronic, autoimmune disease that occurs three times more frequently in women than men and may affect women during their childbearing and childrearing years [13, 14]. Given the early onset of RA and the high prevalence rates in women, reproductive health is a relevant concern for patients and health professionals [15]. Furthermore, living with RA in the first few years after diagnosis can be challenging, [16] and may coincide with, and have important implications for, family planning [11].

There is a longstanding link between RA and infertility with the incidence of nulliparity in women with RA greater than those without the disease [17]. However, lower birth rates may be due to: (i) reduced sexual function [18], (ii) deciding not to have a family, or to have smaller families due to concerns about the impact of RA or its treatment on the foetus [16, 17], or the perception of pregnancy as being risky [19], (iii) fear of transmitting the disease to the child [20]; and (iv) perceived lack of support from their health professionals [12].

It is well established that pregnancy and childbirth can affect the disease activity of RA [21]. During pregnancy a decision to limit the use of RA medication may be made because of the risks that pharmacological treatments can pose to the foetus. However, reduced uptake of disease modifying drugs may lead to increased disease activity in the mother [22]. The development of various biological pharmacotherapies for RA has, for women with RA, supported increased physical capability to manage pregnancy and overcome challenges of raising children [23]. However these therapies may concern women and compound the complexity of their motherhood decision [11].

For women with RA there are some risks associated with pregnancy. While remission can spontaneously occur during pregnancy [24–26] RA symptoms may exacerbate following delivery [27]. Furthermore, there is a link between RA, premature birth, and low birth weight- factors that could have long-term implications for the child’s health [28]. Previous research has identified some of the parenting challenges women with chronic conditions experience, however, little is known about how women with RA make motherhood decisions [11]. It is therefore important that the clinicians discuss with women fertility, pregnancy, and lactations in the context of RA and its treatment, and convey risks and benefits of different options. One approach to facilitating such communication is to provide support for both clinicians and patients, via accessible and targeted educational material in a form of a decision aid tool.

Decision aids (DAs) are based on a well-developed, evidenced-based approach to assisting individuals with making health treatment and screening decisions [29, 30]. The development of DAs is commonly guided by the International Patient Decision Aid Standards (IPDAS) [31]. DAs support active participation in decision making by weighing up the benefits and harms of treatment options based on comprehensive, specific and relevant information [29, 32] from unbiased, nondirective, current research evidence [33].

Results of a comprehensive systematic review based on 55 randomized controlled trial and over 500 DAs conducted by O’Connor and colleagues [34] have found support for the effectiveness of those aids. Stacey et al [32] conducted a recent update of this seminal work to include 115 randomized controlled trials (*n* = 34,444) of DAs. Some of the reviewed DAs refer to decisions regarding pregnancy and birthing options [33]; childbirth options [35, 36], infertility issues [37], miscarriage [38], pregnancy termination [39], and prenatal testing [40]. Another recent systematic review also found positive effects of DAs on informed decision making in pregnancy care, including increased knowledge, and decreased decisional conflict and anxiety [33]. Currently, there is only one published study of a DA on motherhood choices for women with a chronic condition (Multiple Sclerosis) [41].

This paper reports on the randomized controlled study that was conducted to evaluate the effectiveness of a DA developed for women with RA [42]. It was hypothesised that, based on previous DA studies, this DA would (1) increase relevant knowledge and decrease decisional conflict (primary outcomes), and (2) not effect motherhood decision, symptoms of depression and anxiety, or arthritis self-efficacy (secondary outcomes).

## Method

### Ethics

This study was approved (H6884) by the Western Sydney University Human Ethics Committee and conducted according to the Helsinki ethical principles of research. All participants provided written informed consent and were not compensated for their participation.

### Patients and methods

**Fig. 1:**
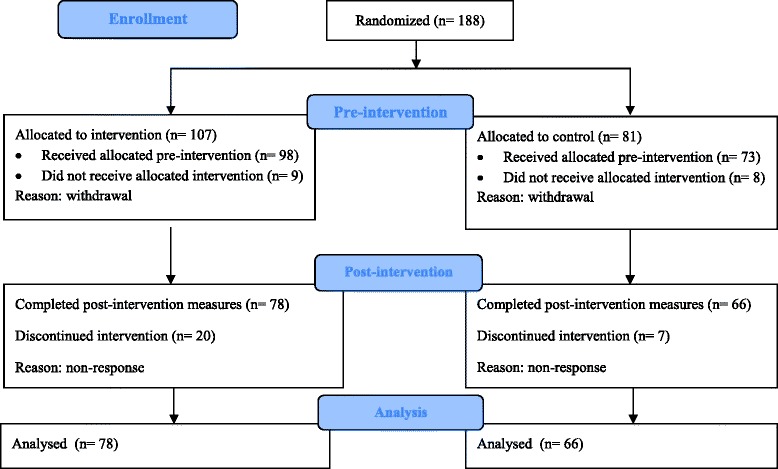
CONSORT flow-chart

The reporting of this randomized controlled study was informed by the CONSORT guidelines [43]. Eligible women were those (i) aged within their child bearing and rearing years that (ii) had been clinically diagnosed with RA and currently under the care of a rheumatologist, and (iii) contemplating having children or more children. No other inclusion or exclusion criteria were applied. One hundred and eighty-eight women enrolled in the study and were randomly allocated to the intervention (DA) or no intervention (control) group (Fig. 1). Of those, 167 women (89 %) completed the pre-intervention questionnaire and 144 women (86 %) the post-intervention questionnaire. Overall, 76 % of women initially enrolled in the study completed both pre- and post-intervention questionnaires.

Recruitment took place over a 12-month period via online advertising including a Google ad campaign, social media, media releases, website content and relevant Arthritis websites, and print advertising including general practitioner newsletters, posters, and flyers distributed to rheumatology clinics. As women provided consent, a member of the research team randomly allocated them to either the DA or control group, using the Bernoulli function in Excel. As a consequence of the random allocation, the control and intervention groups were not balanced for parity or gravity. The pre- and post-intervention questionnaires were completed online via SurveyMonkey.

Once women in the intervention (DA) group had completed the baseline questionnaire, they were sent a link to the electronic version of the DA via email or SurveyMonkey, and were asked to read it within two weeks. All women in the sample possessed an active email account and preferred the DA to be emailed rather than mailed to them. Women in both groups were followed up via phone or emailed approximately two to four weeks after the baseline questionnaire. All women in the intervention group were asked if they had read and understood the DA, and had any questions or feedback about the DA or the study topic. Women in the control group were contacted and asked if they had any questions or feedback about the questionnaire or study topic. Once follow-up contact had been made, women in both groups were sent a link to the post-intervention questionnaire via SurveyMonkey. The questionnaire was identical to the one completed in the pre-intervention phase except for the exclusion of demographic questions. Non-respondents were followed up via phone or email. The process of completing pre and post surveys ranged between four to 12 weeks.

### Measures

The demographic questions included: age, education, marital status, country of birth, RA duration, number of children, and pregnancy status. Participants were also asked whether they wanted children prior to the RA diagnosis, if the diagnosis of RA complicated their decision to have children (*Yes, No,* or *Unsure*), and how certain they were in wanting children in the future (decision certainty) (-5, *Definitely would not have children,* to +5, *Definitely would have children* and a score of 0 indicating *unsure*).

### Primary outcome measures

The *Pregnancy in Rheumatoid Arthritis Questionnaire* (PiRAQ) was developed to assess the RA, pregnancy, and parenting knowledge. Its items correspond directly to the DA content including questions relating to general knowledge about RA, the physical and psychosocial effects of RA, effects of RA on fertility and pregnancy, the effects of RA medicines during conception, pregnancy, and breastfeeding, postnatal considerations, and parenting with DA. Scale development experts were consulted in the development of items, and the final selection was based on pilot results. The PiRAQ was piloted with a consumer sample (women with RA and partners) (*n* = 17). Based on the pilot results the number of items was reduced from 50 to 39 as some lacked discriminatory power. The scores for PiRAQ are calculated by summating the number of correct responses, and range between 0 and 39 with higher scores indicating greater knowledge. In this study, the PiRAQ’s internal consistency coefficient was 0.83.

### Decisional conflict scale (DCS) [44]

The DCS is a 16-item self-report questionnaire which measures personal perceptions of: (1) uncertainty in health-related decision making; (2) factors contributing to the uncertainty and; (3) the perceived effectiveness of decision making. It consists of five subscales: informed, values clarity, uncertainty, support, and effective decision. The scale includes five response criteria ranging from *Strongly Agree* (0) to *Strongly Disagree* (4) with scores ranging from 0 (no decisional conflict) to 100 (extremely high decisional conflict) [44]. The scale has good test-retest reliability (0.81) and internal consistency coefficients range from 0.78-0.89 [44] and in this study was 0.95.

### Secondary outcome measures

#### Arthritis self-efficacy scale (ASES) [45]

The ASES provides a measure of the patient’s perceived ability to control and manage a number of aspects of their arthritis [45]. A short form of the original ASES was used in this study [46]. It contains eight items from the original scale and has been validated [47]. Scores range from 1-10 with higher scores indicating higher levels of self-efficacy. Internal consistency for the short form of the ASES has been reported above 0.90 [46, 47] and in this study was 0.91.

### The hospital anxiety and depression scale (HADS) [48]

The HADS consists of 14 items related to feelings of anxiety (7 items) and depression (7 items). Responses are scored from 0-3, with 3 indicating higher symptom frequencies [49]. Scores may be calculated for each subscale (anxiety and depression) and range from 0-21, with clinical cut off for possible anxiety or depression being a score of 8-10 and probable indication being a score of 11 and over. The scale’s internal consistency coefficients exceed 0.80 [48] and in this study were 0.85. HADS is reported to be suitable for the use with RA populations [50].

### Decision aid

#### Motherhood choices: decision Aid for women with rheumatoid arthritis

Development of the DA was conducted based on the Ottawa Decision Support Framework and following the guidelines of the IPDAS Collaboration [31]. Initial content for the DA was devised based on areas of need identified in the literature. It was then further informed with input from consumer (i.e. women with RA) and expert (i.e. rheumatologists, researchers, health care professionals) panels (*n* = 33). The DA was delivered to the panels via an online survey, with questions pertaining to: content, clarity/readability, balance of information, structure/presentation, and the option to provide qualitative feedback. Panel members also met face-to-face within an advisory committee as part of the feedback gathering process. Statements that were agreed upon by 80 % of the panels’ members were retained, and those with lower agreement rate were revised. Consumer panel members and participants in the pilot study contributed their experiences (‘women’s stories’) to the DA. Pilot testing of the DA, as with the PiRAQ, was conducted with a sample of 17 consumers. In addition to participants completing a battery of questionnaires, they also received a draft version of the DA to comment on its content, clarity, balance of information, and structure. A revised version of the DA was then reviewed by another panel of experts (*n* = 5). Lastly, the Flesch-Kinkaaid readability test was used to ensure that the DA text met the general population’s reading age.

The final DA is a 45-page resource available in electronic or paper versions, and contains three distinct sections: 1) Information on RA, conception, pregnancy, and parenting; 2) decision making activities; and 3) resources. It provides information about RA and its effects, the impact of RA and medications on conception, pregnancy and motherhood, the impact of pregnancy on RA, and ‘women’s stories’. The DA also contains worksheets to assist women in the decision making process (a decision tree, pros/cons scales, support networks, a knowledge checklist, and note taking). The DA is available online on the Arthritis NSW website, http://arthritisnsw.org.au/arthritis/research/ra-and-motherhood/ and is listed on the Ottawa Hospital Research Institute’s Patient Decision Aids website: http://decisionaid.ohri.ca/AZsumm.php?ID=1787. The DA is currently being reviewed based on recent relevant literature.

### Statistical analysis

Analyses were conducted using IMB SPSS Statistics (21.0), with statistical significance set at *p* < .05 (two-tailed). Independent t-tests and ANOVAs were used to assess differences between groups and across time. DAs are reported to increase knowledge by 19 points, and reduce decisional conflict by nine points [51]. A sample size of 130 subjects is sufficient to detect moderate effects (0.13) [52] on each of these differences with 95 % power at a 0.05 level of significance [53]. According to Cohen’s specifications for ANOVA analyses small, medium, and large effect sizes are classified as 0.02, 0.13, and 0.26 respectively [52].

## Results

A total of 188 women consented to participate in the study. Forty-four (28 DA; 16 Control) participants did not complete pre or post questionnaire and after a number of efforts to contact them, were assumed to have withdrawn from the study. The final sample of 144 participants consisted of 78 women (mean age 31.26, *SD* =4.26) in the DA group, and 66 women (mean age 30.67, *SD* = 5.38) in the control group. There were no significant differences on demographic data between women in the DA group, and women in the control group. Demographic data is presented in Table 1.

**Table 1:** Demographic data for 144 participants

Measures	DA group	Control group
	54 % (*n* = 78)	46 % (*n* = 66)
Age (Yrs)	31.26 (4.26)	30.43 (5.07)
Education		
Secondary Education (12 years)	8 % (*n* = 6)	10.8 % (*n* = 7)
Tertiary Education	92 % (*n* = 72)	88 % (*n* = 57)
Marital status		
Single	10 % (*n* = 8)	15 % (*n* = 10)
Married	87 % (*n* = 68)	83 % (*n* = 54)
Divorced/separated	3 % (*n* = 2)	2 % (*n* = 1)
RA Duration in years	8.43 (6.97)	7.71 (7.19)
No. of children		
0	65 % (*n* = 50)	69 % (*n* = 45)
1	26 % (*n* = 20)	23 % (*n* = 15)
2	9 % (*n* = 7)	6 % (*n* = 4)
Pregnant at time of survey	4 % (*n* = 3)	6 % (*n* = 4)
Wanted children before RA diagnosis		
Yes	79 % (*n* = 61)	71 % (*n* = 46)
No	3 % (*n* = 2)	11 % (*n* = 7)
Unsure	18 % (*n* = 14)	18 % (*n* = 12)
Want to have (more) children in the future		
Definitely not	0 % (*n* = 0)	0 % (*n* = 0)
Unsure	51 % (*n* = 40)	38 % (*n* = 25)
Definitely yes	49 % (*n* = 38)	60 % (*n* = 39)

No significant differences were found on demographic data between women in the DA and control groups

Independent samples t-tests revealed no significant differences between the DA and control groups on the PiRAQ, *t*(142) = 1.642, *p* = 0.103, DCS *t*(142) = 1.614, *p* = 0.109, HADS-A *t*(142) = 1.338, *p* = 0.183, HADS-D *t*(142) = 0.764, *p* = 0.446,, or on RA-Complicated Decision, *t*(142) = 1.156, *p* = 0.250 at baseline.

The 2 x 2 mixed between-within subjects ANOVA was conducted to assess the impact of the DA on women’s scores on the PiRAQ, DCS, ASES, HADS-A, and HADS-D, and questions measuring RA-Complicated Decision and Decisional Certainty at follow up (post intervention) (Table 2). Participants in the DA and control groups both had moderate knowledge at pre-intervention, with control group scores remaining unchanged, and the DA group increasing to moderate to high knowledge at post-intervention. Both groups reported relatively low levels of decisional conflict at pre-intervention; however the DA group reported significantly lower decisional conflict at post-intervention. Participants in both groups reported moderate self-efficacy, high levels of anxiety symptoms and low levels of depression symptoms.

**Table 2:** Means, standard deviations, confidence intervals, and effect sizes for each outcome measure at pre and post intervention

	DA group				Control group				ƞp^2^
	Pre		Post		Pre		Post		
	Mean (95 % CI)	*SD*	Mean (95 % CI)	*SD*	Mean (95 % CI)	*SD*	Mean (95 % CI)	*SD*	
PiRAQ	26.70	5.94	31.92	4.66	25.32	4.60	26.09	5.34	0.214**
	(25.49-27.91)		(30.80-33.05)		(24.01-26.62)		(24.88-27.31)		
DCS	43.34	23.92	28.14	18.06	36.96	23.32	34.80	21.86	0.117**
	(38.05-48.63)		(23.69-32.59)		(31.20-42.71)		(29.96-39.64)		
Informed	39.42	26.92	24.57	17.81	44.95	28.34	39.27	27.53	0.036*
	(33.25-45.60)		(19.47-29.67)		(38.24-51.66)		(33.72-44.81)		
Values Clarity	36.54	25.56	25.32	19.62	32.07	27.44	31.06	26.13	0.051*
	(30.62-42.46)		(20.21-30.43)		(25.64-38.50)		(25.51-36.62)		
Support	33.65	23.12	26.82	21.47	31.31	25.19	29.29	20.94	0.014*
	(28.26-39.05)		(22.06-31.57)		(25.45-37.18)		(24.13-34.46)		
Uncertainty	49.89	27.87	40.06	28.52	43.81	28.92	44.82	30.79	0.049*
	(43.55-56.24)		(33.44-46.69)		(36.91-50.72)		(37.63-52.02)		
Effective Decision	32.05	21.75	27.40	21.52	31.06	25.55	30.87	21.28	0.009
	(26.78-37.33)		(22.61-32.20)		(25.33-36.80)		(25.66-36.08)		
Decision Certainty	3.72	1.66	3.51	1.87	3.91	1.72	3.74	1.76	0.014
	(3.34-4.10)		(3.11-3.92)		(3.50-4.32)		(3.30-4.19)		
Complicated Decision	0.91	0.49	0.91	0.50	0.82	0.46	0.96	0.539	0.012
	(0.80-1.02)		(0.79-1.03)		(0.70-0.93)		(0.83-1.08)		
ASES	5.43	1.87	5.81	1.92	5.74	2.00	5.56	2.03	0.030*
	(5.00-5.86)		(5.37-6.25)		(5.27-6.21)		(5.08-6.04)		
HADS-A	8.87	3.73	8.71	3.77	8.00	3.66	8.14	4.13	0.004
	(8.04-9.70)		(7.82-9.59)		(7.09-8.91)		(7.17-9.10)		
HADS-D	5.42	3.41	5.40	3.68	4.94	3.46	5.20	3.92	0.004
	(4.65-6.19)		(4.55-6.25)		(4.10-5.78)		(4.27-6.13)		

*p-value < .05

**p-value < .001

*CI* Confidence Interval, *PiRAQ*: Pregnancy in Rheumatoid Arthritis Questionnaire, *DCS* Decisional Conflict Scale, *ASES* Arthritis Self-Efficacy Scale, *HADS-A* Hospital Anxiety and Depression Scale- Anxiety, *HADS-D* Hospital Anxiety and Depression Scale- Depression

### Primary outcomes

Post-intervention scores on the PiRAQ, revealed a significant time by group interaction, *F*(1, 141) = 38.474, *p <* 0.001, ƞ_p_^2^ = 0.214 (moderate to large effect size), indicating larger improvement in knowledge in the DA group compared to the control group. There was also a significant main effect for group, *F*(1, 141) = 20.787, *p <* 0.001, ƞ_p_^2^ = 0.128 (moderate effect size). Therefore knowledge improved across both groups, but was significantly greater in the DA group.

Decisional conflict (DCS) also reduced significantly more in the DA group than the control group, as indicated by a time by group interaction, *F*(1, 142) = 18.794, *p <* 0.001, ƞ_p_^2^ = 0.117 (moderate effect size). Significant interaction effects were found for three of the four DCS subscales: ‘informed’, ‘values clarity’, and ‘uncertainty’ with all interaction effects being *F*s ≥ 5.28, *p*s ≤ 0.023, ƞ_p_^2^ ≥ 0.036. There was no main effect for group *F*(1, 141) = 0.002, *p =* 0.967, ƞ_p_^2^ = 0.000 but there was a significant main effect for time on the ‘support’ subscale, *F*(1, 142) = 6.719, *p =* 0.011, ƞ_p_^2^ = 0.045. While the effect size was small, this indicates that both the DA and control groups felt more supported in their decision making from pre- to post-intervention.

### Secondary outcomes

Anxiety did not change over time (HADS-A), *F*(1, 141) = 0.004, *p* = 0.947, ƞ_p_^2^ = 0.000, nor differ between groups at post-intervention, *F*(1, 141) = 1.411, *p* = 0.237, ƞ_p_^2^ = 0.010 and there was no interaction effect, *F*(1, 141) = 0.520, *p* = 0.472, ƞ_p_^2^ = 0.004. Depression (HADS-D), also did not significantly change over time *F*(1, 141) = 0.335, *p* = 0.552, ƞ_p_^2^ = 0.003, or between groups, *F*(1, 141) = 0.337, *p* = 0.562, ƞ_p_^2^ = 0.002 with no significant interaction effect, *F*(1, 141) = 0.500, *p* = 0.481, ƞ_p_^2^ = 0.004.

Similarly, there was no significance difference between RA-Complicated Decision from pre to post-intervention, *F*(1, 142) = 1.415, *p* = 0.236, ƞ_p_^2^ = 0.010 across time or groups. Furthermore, the DA did not impact on women’s decision certainty with no significant interaction or main effects (all *F*s ≤ 2.471, *p*s ≥ .152, ƞ_p_^2^ ≤ .014) detected.

For arthritis self-efficacy (ASES), the main effects for time *F*(1, 142) = .550, *p =* 0.460, ƞ_p_^2^ = 0.004) and group (*F*(1, 142) = 0.011, *p =* 0.917, ƞ_p_^2^ < 0.001) were not significant. However, there was a significant interaction effect, *F*(1, 142) = 4.401, *p =* 0.038, ƞ_p_^2^ = 0.030, indicating a post-intervention increase for participants in the DA group, although the effect size was small.

## Discussion

The aim of this randomized controlled study was to evaluate the effectiveness of a DA resource tool developed for women with RA when considering motherhood. In the DA group knowledge increased by approximately 13 % and decisional conflict decreased by 15 % compared to less than 1 % and 2 % respectively in the control group. Similar changes in knowledge and decisional conflict were reported in Prunty et al’s [41] study. While the knowledge increased was lower than the average of 19 % across DA studies [51] this may be due to the already high knowledge at the pre-DA stage. The decisional conflict decline was, however, considerably higher than the average improvement of nine percent [32]. Importantly, the DA did not lead to any increases in psychological distress (anxiety and depression). This confirms that the DA is not associated with negative, unintended consequences as also noted in Prunty et al’s [41] study.

Despite careful attention to the methodology, a few limitations remain, which should be taken into consideration when interpreting the findings. Firstly, reliance on self-selection, and drop out (which in this study was 44/188 participants) effects may have resulted in a sample that is not representative of the broader population of women with RA; particularly in relation to their high rates of tertiary-education, relatively high level of knowledge about RA and pregnancy, and low levels of decisional conflict. Secondly, the evaluation of the knowledge pre and post DA relied on a scale that was specifically developed for this study, as there is no existing RA knowledge scale that addresses pregnancy/parenthood related content, and therefore had limited psychometric evaluation. Thirdly, the DA was self-administered and relied upon self-report data. Fourthly, the short timeframe of the present study means that the usefulness of the DA in actual decision making was not established. Finally, whilst the findings suggest this DA is a useful resource, its effectiveness in face-to-face clinician-patient settings is yet to be evaluated.

These limitations notwithstanding, there are a number of strengths of this study. Firstly, the development of the DA was guided by, and aligned with, the IPDAS criteria [31]. The DA was systematically developed in consultation with both experts and consumers, which informed relevance, clarity, balance, and readability of the DA. It was then piloted and further refined before examining its effectiveness via a randomized controlled study. Lastly, the effectiveness of the DA is consistent with findings of other DAs across a range of health decisions [33, 35]. Given the comparable findings of Prunty and colleagues [41] and the current study it is likely that similar DAs could benefit women with other chronic conditions.

Two issues, however, should be acknowledged. Firstly it is important to note that as there are common concerns and challenges across chronic conditions [11, 54], other DAs may model the core features included in this DA, while generic features, such as value clarifying exercises, may be used in counselling settings [41]. Secondly, despite some similarities, there are considerable differences across chronic conditions (i.e. symptoms, subtypes, treatment, progression, and prognosis). These factors may have unique implications for fertility, pregnancy, and outcomes. Therefore, the existing DAs should not be generalised to motherhood decision making in other chronic conditions.

Given that having children is a decision that may be revisited at different times, a longitudinal evaluation of the impact of DAs is recommended [41] as well as further evaluation of the DA’s usefulness, in terms of its components (particularly in relation to treatment options), across different populations and formats (i.e. briefer, interactive, translated in other languages), and across settings (i.e. clinical, counselling, etc.). Finally, the findings in this study from the DCS’s subscales may be indicative of areas of the DA that could be further strengthened when updating or revising this resource.

## Conclusions

In summary, a DA has been developed to support decision-making for women with RA considering having children or more children. The DA is consistent with the IPDAS criteria, the gold standard for the development of decision aids. This initial evaluation suggests that it is effective in improving relevant knowledge and reducing decisional conflict without influencing women’s decisions or causing distress. This DA therefore has a direct application to patient care in that it may facilitate communication and shared decision making with family and health professionals.

### Availability of supporting data

For access to study data please contact the corresponding author.

## Abbreviations

ASES: Arthritis Self-Efficacy Scale
DA: Decision Aid
DCS: Decisional Conflict Scale
HADS: Hospital Anxiety and Depression Scale
PiRAQ: Pregnancy in Rheumatoid Arthritis Questionnaire
RA: Rheumatoid Arthritis
